# Personalized cloud-based bioinformatics services for research and education: use cases and the *elasticHPC *package

**DOI:** 10.1186/1471-2105-13-S17-S22

**Published:** 2012-12-07

**Authors:** Mohamed El-Kalioby, Mohamed Abouelhoda, Jan Krüger, Robert Giegerich, Alexander Sczyrba, Dennis P Wall, Peter Tonellato

**Affiliations:** 1 Centre for Informatics Sciences, Nile University, Giza, Egypt; 2 Faculty of Engineering, Cairo University, Giza, Egypt; 3 Faculty of Technology, Bielefeld University, Bielefeld, Germany; 4 Centre for Biomedical Informatics, Harvard Medical School, USA

## Abstract

**Background:**

Bioinformatics services have been traditionally provided in the form of a web-server that is hosted at institutional infrastructure and serves multiple users. This model, however, is not flexible enough to cope with the increasing number of users, increasing data size, and new requirements in terms of speed and availability of service. The advent of cloud computing suggests a new service model that provides an efficient solution to these problems, based on the concepts of "resources-on-demand" and "pay-as-you-go". However, cloud computing has not yet been introduced within bioinformatics servers due to the lack of usage scenarios and software layers that address the requirements of the bioinformatics domain.

**Results:**

In this paper, we provide different use case scenarios for providing cloud computing based services, considering both the technical and financial aspects of the cloud computing service model. These scenarios are for individual users seeking computational power as well as bioinformatics service providers aiming at provision of personalized bioinformatics services to their users. We also present *elasticHPC*, a software package and a library that facilitates the use of high performance cloud computing resources in general and the implementation of the suggested bioinformatics scenarios in particular. Concrete examples that demonstrate the suggested use case scenarios with whole bioinformatics servers and major sequence analysis tools like BLAST are presented. Experimental results with large datasets are also included to show the advantages of the cloud model.

**Conclusions:**

Our use case scenarios and the *elasticHPC *package are steps towards the provision of cloud based bioinformatics services, which would help in overcoming the data challenge of recent biological research. All resources related to *elasticHPC *and its web-interface are available at http://www.elasticHPC.org.

## Background

Web-based computational services have gained wide popularity within the bioinformatics community. Bioinformatics service providers have established computational infrastructures, where selected applications of interest are pre-installed and configured according to the system architecture. The model of service provision is that the users interact only with the pre-installed applications through web-interface or web-service. The resources are shared among multiple users according to certain rules and priorities defined by the provider. In spite of the efficiency of this model in facilitating the execution of bioinformatics tasks, both the users and service providers can face a number of limitations due to the rigid design of the infrastructure. As for the users, they cannot use software tools not pre-installed in the system and cannot boost the performance of their tasks by utilizing more computational resources. As for the service providers, it is tedious, complicated, and usually infeasible to scale the infrastructure in response to the increasing computation and data load, which might be abrupt and temporal.

To take one example, Huson et al., [1] discussed the computational challenges in analyzing the metagenomics Sargasso Sea dataset [2]. They indicated that the whole analysis pipeline can run on a moderate infrastructure except for the sequence comparison step based on BLAST, which is the most demanding task that need to be performed on a high performance computer cluster. They estimated that processing the 1.6 million reads of the complete Sargasso Sea dataset would require ≈ 1000 hour on a cluster of 64 CPU. Such intensive computation is a bottleneck for individual users and it is an immense burden for service providers.

Cloud computing is a new form of providing computing services, and in our view it provides a promising solution to overcome the limitations for both the individual users seeking personalized computational power and the computational service providers seeking elasticity of the service. Based on virtualization technology, it enables on-demand allocation, expansion, and reduction of computational resources. It also enables assignment of multiple computing environments with different configurations to multiple users. These services can be offered free of charge or on a pay-as-you-go basis. Currently, there is a number of both academic as well as commercial cloud computing providers worldwide; these include Amazon Web Services (AWS) [3] (which pioneered the provision of such services), Microsoft Azure [4], Rackspace [5], Magellan [6], and DIAG [7], to name a few. The bioinformatics academic community has recognized the advantages of cloud computing [8-12], and the life science industry has started to support its use as well. Interestingly, recent NGS machines can stream the sequenced reads to the client cloud account during the course of sequencing run https://basespace.illumina.com. This means that all the new sequence data become available in the cloud by completion of the wet-lab work.

The features of cloud computing suggests a new model of providing bioinformatics services, where the users do not necessarily share the same environment. Rather, each user can create and configure own infrastructure (machines and storage) and use software tools of interest. This model of use is advantageous to both the bioinformatics user as well as the service provider. The user has the flexibility, better service, and cost saving. The provider no longer worries about scalability and maintenance of resources.

Despite of its advantages, the cloud computing model has not yet been widely used among the community to support bioinformatics services. This can be attributed to two major reasons: First, cloud computing providers offer their services in terms of hardware components (i.e., Infrastructure as a Service, or shortly IaaS) and not in terms of application parameters (i.e., Platform and Software as a Service, or shortly PaaS and SaaS, respectively). This could be a sever barrier for many application scientists who have to dig into many system administration details. Second, there is no well-defined use cases for providing cloud-based bioinformatics servers, considering the platform specifications and the underlying business model. That is, there is a gap between the low-level cloud computing specifications and the application requirements. To bridge this gap, there is a need to 1) develop an efficient middle-ware layer to map the user requirements to low level infrastructure configurations, 2) to define bioinformatics use cases that take technical as well as business details into account.

### Our contribution

#### Models of use

We provide different models of using cloud computing platforms to offer flexible and scalable bioinformatics services. Our models consider not only the technical issues about providing these services, but also the financial aspects, which could be the major concern with respect to the bioinformatics service provider.

The scenarios we suggest are divided into two groups: One for individual users/developers who seek computational power for specific need and one for service providers who wish to provide personalized bioinformatics services. The individual user/developer use cases include the establishment of a computer cluster and running cloud-based jobs either through web, command-line, or programmatic interface. The service provider use case scenarios show how the service provider can scale its resources in case of overload and how a personalized environment can be offered to boost the performance of certain pre-installed tools or the whole system. In these scenarios, we particularly highlight the interaction between the user and the provider server at one side and the interaction between the bioinformatics server and the cloud provider at the other side. We also suggest different options to consider the related financial issues.

#### elasticHPC

We present *elasticHPC *(elastic High Performance Computing), a software package and a library to facilitate the use of high performance cloud computing resources for bioinformatics applications. Although *elasticHPC *is currently based on the Amazon cloud computing platform (Amazon Web Services or AWS), which is the most popular provider of cloud computing services, the concepts presented here are applicable to other cloud computing platforms and this will be achieved in future versions of the library. The basic features of *elasticHPC *include:

• establishment and management of high performance computer cluster (with and without MapReduce framework),

• submission of jobs (from remote site) to the cloud machines and monitoring them,

• establishment of persistent storage in the cloud and linking it to the computing machines, and

• cost management layer to start-terminate jobs based on certain price constraints.

Details of the usage scenarios and their implementation using *elasticHPC *are handled in details in the following implementation section.

### Related technical work

Currently, there are some cloud based programs for bioinformatics applications, especially in the area of analyzing next generation sequencing data. These include, among others, Crossbow [13], RSD-Cloud [14], Myrna [15], and CloudBurst [16]. However, the main focus of these programs was to solve certain problems using parallel infrastructure, and the use of cloud computing was specific to these tools and not of generic nature.

In the time of developing *elasticHPC*, other related software solutions and libraries for AWS have been released. To the best of our knowledge, these include so far StarCluster [17], Vappio [18], and CloudMan [19]. StarCluster [17] was developed as a general cluster management solution for AWS and it is not specific to bioinformatics applications or any bioinformatics use cases. CloudMan [19] was developed as part of the Galaxy project to basically provide a version of the Galaxy workflow system [20,21] in the cloud. (Galaxy is a workflow system developed basically to serve the bioinformatics domain.) CloudMan is not offered as a library but it is offered as a cluster solution with a specific use case scenario. This scenario starts with a creation of a master machine (node) in the cloud from the AWS site using a prepared virtual machine image. From a web-interface on the running master node, the user can add/delete more cluster nodes and start the Galaxy workflow system. Vappio [18], unlike CloudMan, is a standalone library for supporting the creation of a computer cluster in the cloud. It enables submission of remote jobs to the cloud instances. Table 1 shows a comparison between the different features available in the three libraries and *elasticHPC*. (The detailed description of these features is presented in the coming implementation section.) As can be observed from this table, *elasticHPC *includes all the features of both Vappio and CloudMan, and these features along with other unique ones of *elasticHPC *collectively enable the implementation of different use case scenarios for providing personalized bioinformatics services.

**Table 1:** Comparison between *elasticHPC*, Vappio, starCluster, and Cloudman

Feature	*elasticHPC*	Vappio	StarCluster	Cloudman
Create a cloud cluster	Y	Y	Y	Y
Create a MapReduce cluster	Y	N	N	N
Web-interface	Y	N	N	Y
Command line interface	Y	Y	Y	N
Multi-user	Y	N	N	Y
NFS as shared file system	Y	N	Y	Y
S3 as shared file system	Y	N	N	N
Persistent storage after termination	EBS+S3	Manual	Manual	EBS
Data flow model	fetch/shared/replicate	replicate	shared	Shared
Cluster management at run-time	Y	Y	Y	Y
Remote job submission	Y	Y	N	N
Remote job monitoring	Y	Y	N	N
Associated bioinformatics tools	Y	N	N	Y
Use of spot instances	Y	N	N	N

## Implementation

### Amazon Web Services

Amazon Web Services (AWS) is the most popular cloud computing platform. It offers infrastructure as a service (IaaS) in terms of computational power (CPUs and RAM), storage, and connectivity. The AWS products that are of major relevance to solve bioinformatics computational problems include Elastic Compute Cloud (EC2), Simple Storage Service (S3), and Elastic Block Storage (EBS).

EC2 includes a variety of user selectable machine instance types that range in computing power and cost. Table 2 summaries the features of some instance types including the strongest ones. With each of these types, mounted disks (called ephemeral disks) are also provided. Virtual machine instances are created from Amazon Machine Images (AMI), which are templates containing software configurations (e.g., operating system, application server, and applications). To facilitate the creation of virtual machine instances, EC2 includes a directory of AMI's either prepared by AWS or by the community. This directory includes a variety of AMI's with different operating systems and additional applications. From the AWS web-interface, the user can set-up, launch, terminate any number of instances within a few minutes.

**Table 2:** Amazon Services: virtual machines, storage, data transfer, and disk access (US-East site)

Resource Type	AWS Service	Service Unit	CPUs (#(GHz))	Memory (GB)	Cost($/hr)
		m1.large	2 (2)	7.5	0.32
		m1.xlarge	4 (2)	15	0.64
Computation	EC2	c1.xlarge	8(2.5)	7	0.66
		m2.4 × large	8 (3.25)	68.4	1.80
		cc1.4 × large	8 (4.19)	23	1.30

**Resource Type**	**AWS Service**	**Service Unit**	**Size**	**Tiers**	**Cost ($/GB/Month)**

	S3	Bucket	unlimited	1^st ^1 TB	0.14
Storage	S3	Bucket	unlimited	Next 450 TB	0.1
	S3	Bucket	unlimited	Next 4000 TB	0.08
	EBS	Volume	Up to 1 TB		0.10

**Resource Type**	**AWS Service**	**Service Unit**	**Type**	**Size**	**Cost ($/GB/Month)**

	S3	I/O	Data IN/Within AWS	Any	0.00
	S3	I/O	Data OUT	1st 1 GB	0.00
Data transfer	S3	I/O	Data OUT	Next 10 TB	0.12
	S3	I/O	Data OUT	Next 100 TB	0.07
	S3	I/O	Data OUT	Next 150+ TB	0.05

	S3	API	GET/PUT/POST	1 K Requests	0.01
Data Access	S3	API	COPY/LIST	1 K Requests	0.01
	EBS	I/O	R/W	1 M Requests	0.1

Because the ephemeral disks are volatile and vanishes with the termination of the machine, AWS offers two types of persistent storage: EBS and S3. The former is defined in terms of volumes, where one or more EBS volumes can be attached (mounted) to a running instance, similar to a USB thumb drive (volume size ranges from 1 GB to 1 TB). The latter is like a data center accessed through certain programmatic methods.

The AWS business model is "pay-as-you-go", where the user is charged only when the machines are running. The user is also charged for reserved storage and for data transfer out of the AWS site and from/to persistent storage solutions. Table 2 summarizes the storage options and their prices in AWS (price update of March 2012). For more information about the AWS pricing schemes, we refer the reader to the documentation available on AWS web-site [3].

In addition to the web-based interface, AWS's services can be accessed programmatically through command line interface and AWS-specific API's. We use programmatic methods in *elasticHPC *and build upon them to provide an efficient implementation and an easy to use interface for the use case scenarios presented below in this section.

### Use case scenarios

As mentioned in the introduction, we suggest two groups of use case scenarios: one for individual users and one for bioinformatics service provider. In this part of the paper, we discuss these scenarios and their implementation using *elasticHPC*.

#### Use case scenarios for individual users

Use case scenarios for individual bioinformatics users have been implicitly introduced with Cloudman and Vappio. For completeness of presentation, we will discuss these scenarios. Then we will show the major differences between them and suggest further usage and implementation enhancement. The suggested use case scenarios for individual users involve two cases: 1) Web based usage, and 2) desktop based usage.

##### Scenario 1: web-based usage

In this scenario, a bioinformatics user, who already has an AWS account, heads for establishing an infrastructure composed of a computer cluster with multiple machines of certain types in the cloud. The major steps for achieving this task include the following:

1. A third-party development team prepares a virtual machine image, equipped with necessary bioinformatics tools and middleware to create and manage a computer cluster. This machine is then deposited in the public AMI's repository of AWS. The bioinformatics tools can be augmented with the associated web-interface.

2. The bioinformatics user who wishes to run an application in the cloud selects that image from the public AMIs page of AWS.

3. A machine instance is created from the selected AMI and is kept running. This machine is the master node of a cluster.

4. The IP address of the running machine is retrieved from the Amazon console, and the machine is accessed remotely by one of two means:

a. Secure shell, where any further interaction with the cluster takes place at the command line level.

b. Web-interface, where the machine includes a web-server that receives requests and processes them. Each computational tool can have its own web-page to set related parameters and enter the input.

5. Based on installed API's on the master node, the user builds and configures a computer cluster in the cloud with the desired number and types of machines instances. The rationale behind that a cluster node creates other nodes is twofold: 1) The AMI contains all the required and tested modules with all settings and technical details being encapsulated, which reduces the user's effort and cost. 2) All the steps run in parallel at the AWS side with no further communication overhead.

6. Storage in the form of S3 or EBS products is attached to the cluster nodes.

7. The execution of tasks can proceed through the master node using either command line or web interface. Note that we mean with the task any job running in the cloud; i.e., installation of a software program is also considered tasks.

**Figure 1:**
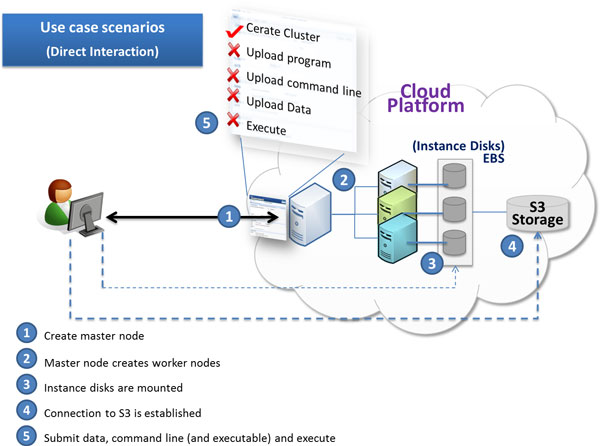
**Use case scenario for individual users**. Use case scenario for individual user: Dotted lines correspond to data flow, where the user uploads/downloads his queries/databases.

Figure 1 shows the steps of this scenario. The user can start execution only when the cluster nodes are created. The establishment and usage of storage is handled later in the storage and data flow subsection.

##### Scenario 2: desktop based access

In this scenario, the user installs a desktop application (developed by a third party) that automatically connects to AWS, spots a prepared AMI, and starts it. From the desktop interface, the user specifies the size and type of cluster and storage. The desktop communicates with the master node to extend the cluster, if required. The submission and monitoring of the jobs is achieved through this desktop application via the master node.

The implementation of the two scenarios is very similar except that an additional piece of code should be developed in the second scenario to realize a communication protocol between the desktop program and the cloud machines. It is worth mentioning that Cloudman follows the first scenario, while Vappio follows the second scenario.

##### Data flow

The data flow for both scenarios involve the transfer of user data to the ephemeral disks, EBS, or S3. It also involves how the data are made available to the cluster nodes. There are four data flow scenarios: In the first one, each cluster node receives a copy of the whole dataset either in the ephemeral disks or EBS mounted volumes. (Ephemeral disks are volatile, and the user should have a warning to move the data to persistent storage.) In the second scenario, a shared file system like (NFS) is installed, where the nodes share the whole disk space. In the third scenario, S3 is used as a shared file system. In the fourth scenario, each node fetches the data it needs from the master node or S3 bucket before starting computation. Vappio adopts the first data flow scenario, while Cloudman and StarCluster adopt the second scenario.

##### Enhancement

*elasticHPC *enables creation of EMR cluster and use of EMR framework. This feature is not available in any other library. It also enables use of spot instances in AWS, where the cluster starts only if the machine price falls below a given threshold. *elasticHPC *provides the four data flow scenarios, and not limited to just one strategy. It also provides an enhanced implementation of the first data scenario based on replication of data, where the copy operation proceeds in parallel in *O*(*n *log *P*) time, *n *is the data size and *P *is the number of nodes.

#### Use case scenarios involving service provider

We suggest two main use case scenarios involving a service provider. These scenarios address not only the technical but also the financial part of the model, where the major concern of the providers is "To which account will the cloud cost be charged?"

##### Scenario 1: bypassed provider

This scenario includes the following steps:

1. The bioinformatics service provider prepares a machine image of his system.

2. The provider offers a web-page, where the user defines cloud based computer cluster in terms of machine type and number. The cluster will be established using images that have been prepared by the provider. The provider can keep track of the users and store their preferred cluster configurations.

3. The user provides its cloud computing credentials so that the created cluster is associated with the user account; i.e., the cluster runs at the cost of the user, and the provider account is not charged whatever computation time is elapsed.

4. After the creation of the machines, the user is directed to the cloud version of the provider system, where all tools are available with the usual web-interface.

5. The provider provides a cluster management page in the cloud version where the user can manage the cluster at the run time and can also terminate it after completion of computation.

##### Scenario 2: Usage-via-provider

In this scenario, the computer cluster in the cloud is hidden from the user, and every step runs through the main web-site of the bioinformatics service provider. This scenario includes the following sequence of events.

1. The user opens a (tool) page on the provider site, and selects that the execution takes place in the cloud. In this case, the user is prompted to configure and to create a new cluster or select one of running clusters (if any exist). Once the cluster is created, the respective tool in the cloud image of the cluster is the one that is invoked.

2. The data, if not residing in S3, could be uploaded to the cloud cluster by one of two means: First, the data is uploaded to the provider, who forwards it to the cloud. Second, the data can be uploaded directly to the cloud through a special client side script installed at the user machine. The second method has the advantage of reducing traffic at the provider site.

3. The provider monitors the progress of the job and the user follows this progress from the provider web-pages.

4. After completion, the provider could buffer the data to be downloaded from the tool page or provide links so that the user downloads the results from the cloud. The latter scenario reduces traffic at the server site.

**Figure 2:**
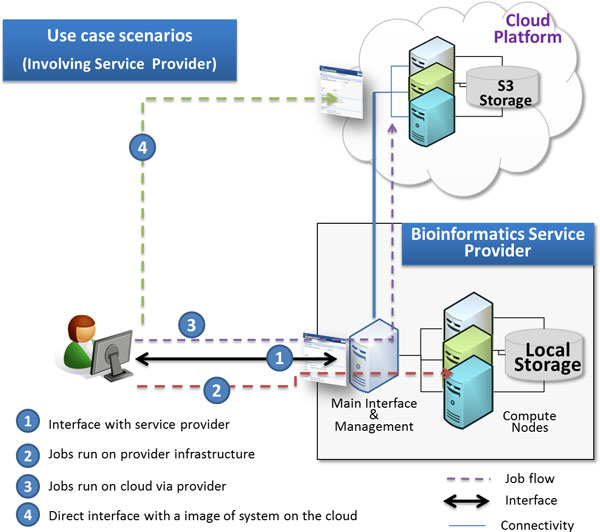
**Use case involving bioinformatics service providers**. Use case scenario showing the interaction between the bioinformatics service provider and the user at one side and between the service provider and the cloud provider at one the other side.

**Figure 3:**
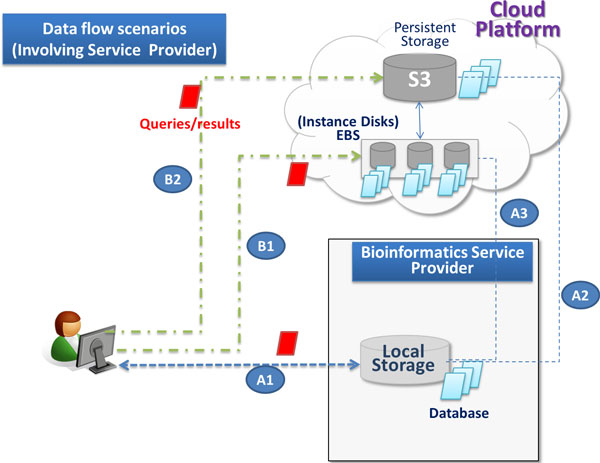
**Data flow scenarios**. Data flow scenarios involving service provide. In step A1, the user sends data to the provider. Provider, according to user options, sends data either to EBS volumes as in step A2 or to S3 as in step A3. In steps B1 and B2, the user directly sends data to the cloud version and receives the result without passing through the provider.

Figures 2 and 3 show the traditional way of running jobs on provider infrastructure and show these cloud based scenarios.

There are some issues related to these two scenarios:

• The first scenario is usually offered to users that are considered foreigner with respect to the provider. The computation runs at the cost of the users and the provider is not charged whatever computation time is elapsed and whatever data is transferred. In the second scenario, the cost of using the cloud can be taken over by the user (if the user's credentials are submitted) or by the provider.

• The second scenario can be used when the provider wishes to scale up infrastructure in a hidden manner in response to an increasing server load. Here, the user requests are forwarded to the cloud cluster and the results are provided back to the user through the server web-interface.

• The second scenario can help those users that are related to the provider and have no cloud account to use the cloud based services. The cloud server runs at the cost of the provider, who can manage the consumption of each user through one of two scenarios:

1. The provider uses a user management layer to control the computation for each of such users. For example, each user could be dedicated some free computation time. The provider controls the cost by starting and terminating the machines according to the available user credit.

2. The provider associates his account with the user account such that the provider takes over the computation cost until certain amount. This is achieved through the consolidating billing option in AWS.

• The bioinformatics service provider can offer an additional service in which the snapshots of the databases needed by the users are made accessible to them. The cost of maintaining the snapshots in the cloud is covered by the provider. These snapshots can be used to create any number of EBS volumes to be used in computation.

• For each scenario, the provider can offer the whole system in the cloud or just some tools. When the focus is on using a certain tool in the cloud, further abstraction and optimization can be offered to the user. The idea is that the provider, based on his log data and knowledge of tool characteristics, can provide the service in terms of application parameters and not in terms of hardware parameters. For example, if the program in use were BLAST, then the user could be asked to specify the cost limit or the desired time by which the computation is over, in addition to other information about amount of data, its type (DNA, RNA, or protein), the BLAST program (blastp, blastx, blastn, etc.). The provider, based on these parameters, could then create the suitable infrastructure to accomplish the given computation task.

• The second scenario at the tool level can support execution of pipelines, where a part of the pipeline is executed at the cloud and the other parts at the provider's local infrastructure. This pipelining can be done through pipeline/workflow authoring interface at the provider site. The cost of cloud in this case can be taken over either by the provider or the user, when the latter submits own credentials.

**Figure 4:**
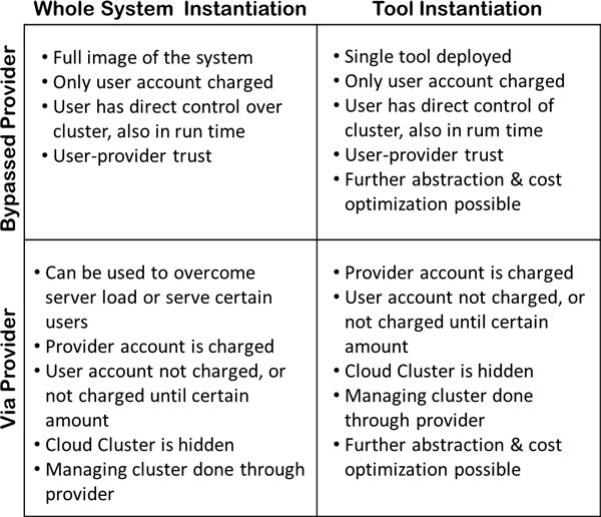
**Features of each use case**. Features of each use case involving provider with whole system or a tool being instantiated in the cloud are arranged in a matrix view.

Figure 4 shows a matrix that summarizes the properties of each of these scenarios, both on the system and tool instantiation levels.

### *elasticHPC *design and implementation

**Client-server based approach: **the *elasticHPC *package is composed of a server and a client modules as well as a set of methods for creating and managing the cloud cluster and for running jobs. The server and client are written in Python. To save user's time, we already prepared a machine image and pre-installed the *elasticHPC *package in it. The security settings are pre-configured within the machine images. That is each machine instance works as both a client and a server. The idea to have a server module on each node is to enable execution of a task (including any administration job) on this node from another remote node. An asynchronous protocol is used to enable the client module to execute different tasks on the server side. The client module is standalone and can be installed on any machine, for example the user's local desktop, to submit jobs to the cloud cluster. The tasks executed on the server can be broadly categorized into the following two categories:

• Cloud-oriented tasks: these include functions for the establishment of additional cluster nodes and terminating some of or all the running ones. It also includes the configuration of the nodes and the set-up of the network connectivity, the storage, and job-scheduler. These tasks are accomplished by executing special *elasticHPC *programs which are pre-installed on the server machine. These programs invoke AWS API's to run the cloud related functions and invoke other scripts to administrate the nodes. The creation of the master node takes up to 2-3 minutes for a single node. The creation (including configuration) of the other cluster nodes is accomplished in parallel and it takes similar time per node. By means of this parallelization, the total time for establishing a cluster of any size ranges between 2-6 minutes.

• Computation-oriented tasks: this includes the reception of a job from a client program and executing it on the computer cluster. This job is basically an invocation of a program already installed on the cluster. The user can submit the input data and fix the location of the output in the cluster. The server can also report the status of submitted job to the client. Note that the user can invoke an installer program as a job to install other programs.

**Included middle-ware and libraries**: an *elasticHPC *image is based on Linux Ubuntu and it is equipped with a number of programs to facilitate its usage. These include Amazon Command Line Tools, Apache server, PBS Torque as a job scheduler, NFS as a shared file system, s3fs [22] to handle the S3 as a shared file system, the boto library http://code.google.com/p/boto, Python/Perl interpreters, MPICH2, and C/C++ and Java libraries.

## Results and discussion

### *elasticHPC *features and distribution

**Figure 5:**
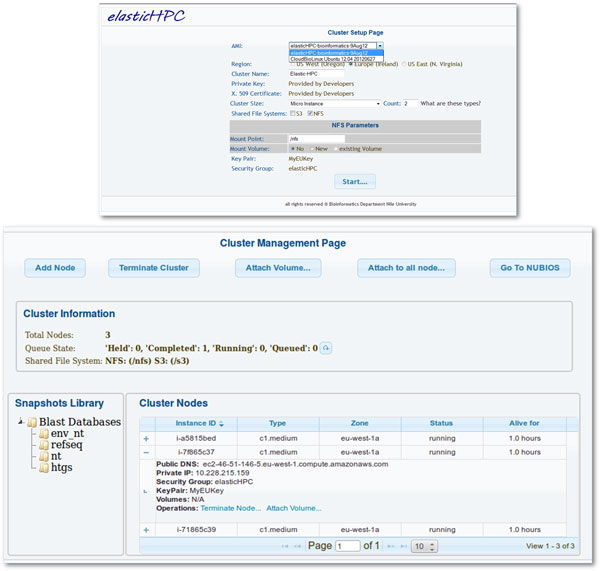
***elasticHPC *set-up and management pages**. *elasticHPC *set-up and management pages. The set-up page is accessed from the *elasticHPC *web-site. The provider can use a similar page in his web-site to start the personalized cloud service. In the set-up page, the user can set the number and type of cluster machines. In the management page, the user manages the established cluster. Nodes can be added or deleted. Status of each node can also be monitored. Storage volumes can be mounted to one or all the machines. Clicking on the BiBiServ-NUBIOS link takes the user to the web interface pages of the hosted bioinformatics tools from the BiBiServ and NUBIOS systems.

The *elasticHPC *package and library supports the use case scenarios presented in this paper. The distribution includes the library source code and full documentation of its entire API's. For individual users wishing to exploit cloud computing, we prepared a machine image with the *elasticHPC *package pre-installed in it with additional features described below. This image can be directly used from Amazon AMI directory as given in the manual. The running image has a simple web-based user interface on the master node to manage the cluster. This interface enables management of the cluster and submission of tasks. This image can also be started from the *elasticHPC *website http://www.elastichpc.org where the user can set-up the cluster; note that in this case the *elasticHPC *website represents a service provider. Figure 5 shows screen shots for the set-up in the *elasticHPC *website and the cluster management page in the master node of the cloud cluster. The provider can use a similar set-up page in his web-site to start the personalized cloud service. In the set-up page, the user can set the number and type of cluster machines. In the management page, the user manages the created and currently running cluster. Form this page, nodes can be added or terminated and storage volumes can be mounted to one or all the machines. Status of each node can also be monitored.

**Figure 6:**
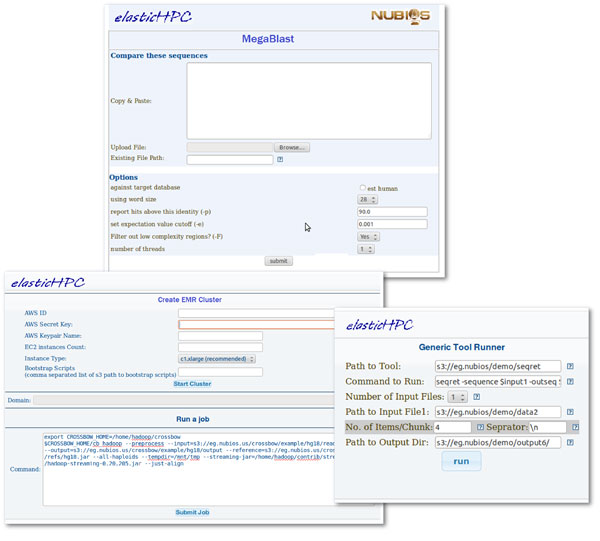
**Invoking tools for traditional and EMR cluster**. The web-interface page shows MegaBlast tool where the tool parameters are set. The user enters the path to the data on the S3 account or uploads the data directly to the tool. The lower left screen shot include the web-interface of using the elastic MapReduce service (EMR), where the user specifies the EMR cluster, initialization scripts, and command line to be executed, as explained in the manual. The lower-right screen shot is for generic submission of jobs on traditional cluster. In this case, the user has own software tool (in compatible and executable format), which resides in the cloud. The user specifies the location of this tool, specifies the command line, and specifies the input and output data. This web-page enables execution of this tool on the system. More details are given in the manual.

The *elasticHPC *image/machine has additional features that leverage the use of cloud to the bioinformatics domain. These include the following:

• The *elasticHPC *image includes a set of pre-installed tools that can be directly accessed upon the creation of the cluster. In the current version of *elasticHPC*, there are about 200 tools, coming from BioLinux, EMBOSS [23], SAMtools [24], fastx [25], NCBI BLAST Toolkit [26-28], and other individual sequence and RNA analysis programs. These tools come from the BiBiServ and NUBIOS bioinformatics servers. Addition of extra tools and updating this image is explained in the *elasticHPC *manual. In the management page in Figure 5, the button titled 'BiBiServ-NUBIOS' let the user move to the library and the web-pages of individual tools. Figure 6 shows a screen shot of the MegaBlast web-interface on a created cluster in the cloud. In this Figure, we also show the web-interface for generic tool submission, where the user can run a tool not pre-installed on the cloud. From this page, the user gives location (S3 path) of the tool binaries, specifies the input and output files, and runs the tool. In this way, the use of this library is not limited to any pre-defined set of tools.

• To save user's cost and to facilitate usage of database-dependent programs, we have prepared snapshots of different databases. These snapshots are made available to the user free of charge through a simple user interface, to create EBS volumes and mount them to the cluster. Our snapshots currently include the NCBI nucleotide and protein databases in the form of raw and formatted sequences. These also include the raw human genome sequence, and pre-computed indexes of it to be used with some tools, as explained in the manual. In the management page in Figure 5, the snapshot list on the left includes identifiers for these volumes to be attached to the cluster nodes.

• The user has the option to select the location, where the cluster is hosted. Our benchmark data (not included in this manuscript) has revealed that the location of the user affects the latency and quality of the service. We found that the AWS European site, for example, is the one with the reduced latency for south Mediterranean countries. For Asian countries, the AWS Singapore would be the best choice. Furthermore, the time of acquiring the service also plays a role; e.g., the US sites have the highest load on Monday (especially in the morning), so it would be a good idea to switch to other sites. Note also that some machine instance types are offered in one site but not in the others; e.g., cluster type is so far available only in the US East site.

• *elasticHPC *offers the creation of a MapReduce cluster. This option facilitates the use of certain bioinformatics tools, like Crossbow [13], RSD-Cloud [14], Myrna [15], and CloudBurst [16]. From the *elasticHPC *web-site the user can set-up a MapReduce cluster and submit jobs to it. Figure 6 shows a screen shot of these two steps, where the user specifies the cluster, defines initialization scripts, and the hadoop command line, as explained in the manual. The example in the Figure is for the program Crossbow [13], which is already included in the *elasticHPC *image.

• Clusters built by *elasticHPC *can be used by multiple users. The jobs and their input and output data are associated with user identification numbers. This feature is helpful for service providers extending their infrastructure for multiple users.

• *elasticHPC *supports different cloud storage services, like ephemeral disks, S3, and EBS. Data are made available to a cluster node in *elasticHPC *through one of three options: 1) Data replication, 2) Sharing, and 3) Fetching. In data replication, the user can broadcast the data (queries and databases) to all cluster nodes. In the sharing option, the disk space is shared among all nodes using a shared file system (NFS). *elasticHPC *offers the use of NFS and S3 as shared file systems. In the final mode, *elasticHPC *can fetch necessary data for execution (if not available) from the master node or S3 before execution of a command. Each of these scenarios and storage solutions is advantageous for certain applications and certain types of data. The access to S3 is more reliable but it is slower and more expensive. (Recall that AWS charges for each S3 request.) The use of NFS over ephemeral disks or EBS volumes is better and more cost effective than using S3 as shared file system, but it is less reliable, as discussed in [29]. Ephemeral disks are as efficient as EBS volumes, but ephemeral disks are volatile and data have to be moved to them before computation and out of them after computation. Data copy option over ephemeral disks or EBS volumes is the most efficient option with respect to reliability and run-time execution, but the disadvantage is that 1) some data might not be used at all in some cluster nodes, which lead to unnecessary disk and I/O cost, and 2) the data could be very large and take much time to transfer. The manual of *elastiHPC *includes more details on the use of these storage solutions and related dataflow scenarios.

• *elasticHPC *enables further cost management where the user starts the cluster only when the price of an instance falls under certain threshold in AWS. This option is based on the use of spot instances of AWS.

• *elasticHPC has an additional API to start other machine images such as CloudBioLinux *[30]http://cloudbiolinux.org, *which encapsulates BioLinux and CloudMan. (CloudMan includes the Galaxy workflow system.) This option is not specific to these images and can be used with other AMIs. This feature is inspired by the BioCloudCentral project *https://biocloudcentral.herokuapp.com*aiming at providing a central point of access to start different AMI's*.

### Overview of demonstrations and experiments

In the following subsections, we introduce demonstrations of individual user scenario and bioinformatics provider scenario. The individual user scenario includes two experiments: The first is a metagenomics experiment based on MegaBLAST to demonstrate the use of a traditional computer cluster. The second is an experiment for mapping NGS reads to a reference genome using Crossbow to demonstrate the use of MapReduce framework on AWS. The provider scenario demonstrates the use of cloud services on the tool as well as the whole system level, as implemented in the Bielefeld Bioinformatics Server (BiBiServ) and the Nile University Bioinformatics Server (NUBIOS).

### Demonstrating individual user scenario

#### Demonstrating the use of traditional computer cluster

To demonstrate the efficiency of the cloud model, we used the *elasticHPC *web-interface from its web-site http://www.elasticHPC.org to construct a cluster of 4 nodes to analyze metagenomics datasets. The MegaBLAST [28] program is used to annotate a set of sequences coming from a metagenomics experiment. With this cluster, we mounted EBS volumes including the NCBI NT database. Figure 6 shows the web-interface of the program MegaBLAST [28] on a cluster machine in the cloud.

We used the windshield dataset of [31], which is composed of two collections of 454 FLX reads. These reads came from the DNA of the organic matter on the windshield of a moving vehicle that visited two geographic locations (trips A and B). For each trip A or B, there are two subsets for the left and right part of the windshield. The number of reads are 66575 (12.3 Mbp) 71000 (14.2 Mbp) 104283 (18.8 Mbp) 79460 (12.7 Mbp) for trips A Left, B Left, A Right, and B Right, respectively.

To make use of the parallel infrastructure, we enhanced the web-interface with an extra function that splits query set into multiple subsets, each containing roughly the same number of sequences. These subsets are then queried in parallel against the MegaBLAST database based on the installed job scheduling system. For the dataset at hand, we created 11 subsets, each with ≈ 6000 reads. For this experiment, an EBS volume including the required databases was mounted to each cluster node. To make the query data available to each cluster node we used the fetch strategy, in which each node fetches the queries assigned to it from the S3 directory before starting computation.

Table 3 shows the average running times over computer clusters of different machine types and different number of compute nodes. The machine types used include nodes of the type "Extra Large High CPU" and of the type "Extra Large High Memory". The establishment of any of those clusters with the storage took a few minutes from the machine images. Table 3 also includes the monetary cost of running these experiments. It is interesting to see that the use of more machines led to faster running time with the same or reduced cost. The best price was obtained with 8 cores. The fastest time was obtained with total 32 cores, which ran for less than one hour and cost totally $2.7. We also note that the use of machines of the type m1.xlarge lead to more running time and more cost than the use of the same number of machines of c1.xlarge. This is although m1.xlarge is less expensive than c1.xlarge.

**Table 3:** Running times of the metagenomics experiment in the cloud

Dataset	AWS Cores				
	
	1	8	16	32	64
c1.xlarge (8 cores)					
	1 node	1 node	2 nodes	4 nodes	8 nodes
Trip A Left	93 ($1.32)	27 ($0.66)	20 ($1.32)	13 ($2.64)	9($5.28)
Trip A Right	127 ($1.98)	33 ($0.66)	21 ($1.32)	13 ($2.64)	7($5.28)
Trip B Left	80 ($1.32)	25 ($0.66)	17 ($1.32)	13 ($2.64)	7($5.28)
Trip B Right	65 ($1.32)	23 ($0.66)	13 ($1.32)	8 ($2.64)	6($5.28)
Total	365 ($4.62)	108 ($1.19)	71 ($2.64)	47 ($2.64)	29($5.28)

m1.xlarge (4 cores)					
	1 node	2 nodes	4 nodes	8 nodes	16 nodes
Trip A Left	77 ($1.28)	18 ($0.64)	13 ($2.64)	9 ($5.12)	7($10.24)
Trip A Right	119 ($1.28)	34 ($0.64)	25 ($2.64)	16 ($5.12)	10($10.24)
Trip B Left	70 ($1.28)	31 ($0.64)	23 ($2.64)	15 ($5.12)	9($10.24)
Trip B Right	65 ($1.28)	27 ($0.64)	13 ($2.64)	9 ($5.12)	6($10.24)
Total	331 ($2.56)	110 ($1.28)	74 ($5.12)	49 ($5.12)	32($10.24)

The average running times in minutes for traditional computer clusters of different sizes and machine types in the cloud. The machine types are c1.xlarge and m1.xlarge. The numbers in brackets are the computation costs in US Dollar for the US-East site with $0.66 per hour for c1.xlarge and $0.64 per hour for m1.xlarge. (Note that partial computing hour of an instance is billed on Amazon as a full hour) The cost in the column titled "Total Time" is not the summation of the above rows, but it is the cost of the total running time if the four datasets in the respective column were processed altogether in the cluster.

#### Demonstrating the use of MapReduce

*elasticHPC *supports the use of MapReduce framework on AWS, where the user can create EMR cluster on AWS by specifying the number of nodes and machine types. The EMR cluster, despite being more difficult to use than job schedulers, has the advantage of lower machine price in AWS. The usage of EMR framework within *elasticHPC *is generic to any tool as explained in the manual. To further facilitate the work for life science community, we included the Crossbow [13] tool within the *elasticHPC *distribution. To demonstrate the advantages of EMR, we ran an experiment to map a dataset of NGS reads to a reference human genome using Crossbow. The dataset is composed of illumnia reads of around 13 Gbp (47 GB) from the African genome available at the Sequence Read Archive (http://trace.ddbj.nig.ac.jp, SRR002271 to SRR002278). The reads of this dataset were mapped against the human genome (hg18, build 36) available at the UCSC Genome Browser web-site http://genome.ucsc.edu.

We used EMR clusters of different machine types and number of nodes. Table 4 shows the running times of these experiments and their monetary costs on Amazon EMR. The results show that Crossbow over EMR scales well with the increasing number of machines. The limit is the pre-processing step of Crossbow, which cannot take less than ≈33 minutes.

**Table 4:** Running times of Crossbow on EMR using *elasticHPC*.

Num Nodes	Num Cores	Processing Time	Mapping Time	Total Time	Cost
Using c1.xlarge					

1	8	66 m	769 m	835.1 m	$1.68
4	32	39.5 m	258.6 m	298.4 m	$2.4
8	64	35.25 m	121.5 m	156.8 m	$2.88
16	128	34.1 m	62.6 m	96.9 m	$3.84
24	192	33 m	46.6 m	79.8 m	$5.76
32	256	33.0 m	39.95 m	73.5 m	$7.68
64	512	32.65 m	23.6 m	56.1 m	$7.68

Using m1.xlarge					

1	4	72.2 m	1675.6 m	1748 m	$2.7
4	16	40.6 m	431.4 m	472.6 m	$2.88
8	32	37.3 m	263.8 m	301.1 m	$4.32
16	64	33.6 m	95.6 m	129.6 m	$4.32
24	96	32.9 m	54.2 m	87.1 m	$4.32
32	128	32.6 m	51.5 m	84.3 m	$5.76
64	256	32.8 m	33.3 m	66.1 m	$11.52

The average running times in minutes for EMR clusters of different sizes and machine types in the cloud. The machine types are c1.xlarge and m1.xlarge. A number in the column titled 'Total Time" is the summation of the pre-processing and alignment times.

It is worth mentioning that we ran the same experiments using a job scheduler on non-EMR clusters of corresponding sizes. The experiments took almost the same time, but with much higher cost. This is because the cost of a traditional cluster node is more expensive than the cost of a node of the same type in the EMR product. For example, the price of a node of the type c1.xlarge is $0.64 per hour, while the cost of a node of the same type in EMR product is $0.12 per hour. (Price for a node of type m1.xlarge is $0.09.) That is, the use of EMR cluster is very cost-effective; of course only if the problem at hand can be formulated according to the MapReduce framework. Note that the cost of using 8, 16, or 24 m1.xlargre nodes is $4.32, because in all these cases there are 48 compute hours, each with a cost of $0.09. Note that the use of more than 64 nodes will lead to more cost with no significance reduction in running time. This is because a fraction of an hour is charged as a full hour in AWS. We also note that the use of machines of the type m1.xlarge mostly lead to more running time and more cost. This is although m1.xlarge is less expensive than c1.xlarge.

### Reliability of the cloud based model

For our use cases, reliability of computation on cloud in our view can be addressed at two levels: The first is the ability to acquire required resources, including machine instances, storage, and connectivity. The second is the ability to successfully execute compute jobs on the cluster machines.

The reliability of acquiring resources was limited by the contention of network bandwidth and the highest load, especially at US sites. In the last few years, the reliability of AWS is dramatically increased by establishment of many sites (locations) worldwide and continuous improvement of the underlying cloud software stack. Before the establishment of US-West site in 2011, for example, users including our team have observed highest latency at US-East site and some failure of acquiring network resources for their (EMR) clusters. We expect further improvements when more sites are established worldwide.

Reliability of computation with respect to the use case scenarios, presented in this paper is associated with the use of the traditional and EMR computer cluster. Failure of certain nodes and the inability of transferring data among the nodes are the most two concerns in cloud settings. In our cloud based experiments involving tens of nodes, we did not experience such type of errors so far for traditional computer cluster. For EMR, we had encountered such problems only before Amazon upgraded the Hadoop version to 0.20.205 in EMR and tuned its performance. But such errors could appear when using hundreds and thousands of nodes. Although the job scheduler and Hadoop implementation of EMR address the failure issues by re-directing jobs failed on one node to other running nodes and by replication of data on multiple nodes, we think there is a need for another layer built upon *elasticHPC *to assure that the data required by the re-directed jobs is available in the new node and to track the jobs that completely failed for some reasons. This layer can make use of the *elasticHPC *feature of reducing cluster size in run time to re-execute failed jobs with smaller cluster to save further cost. Altogether, handling fault tolerant computation in the cloud is a topic of current research and it deserves to be addressed separately in another study.

### BiBiServ-NUBIOS: demonstrating service provider scenario

BiBiServ http://bibiserv.techfak.uni-bielefeld.de is a bioinformatics server hosted at Bielefeld University. The server focuses on RNA sequence analysis, and it includes tools for secondary structure prediction of RNA (e.g., RNAfold [32], RNAshapes [33]), comparative structure prediction (e.g., RNAalifold [34], Foldalign [35]), and RNA structure comparison (e.g., RNAforester [36]). The user can just use one tool or use the tools in a pipelined fashion. This pipelined mode became possible only through consistent data types as specified in [37] and through the storage of intermediate data on the server in each usage session.

NUBIOS http://www.nubios.nileu.edu.eg is a bioinformatics server hosted at Nile University. The server focuses on sequence analysis tasks and hosts tools from EMBOSS and NCBI toolkit. The web-interface of the tools is generated on the fly, according to certain XML-based schema describing the interface components and the tool parameters. The tool set in NUBIOS complements that set of BiBiServ and it is useful to have both systems accessed in the cloud.

**Figure 7:**
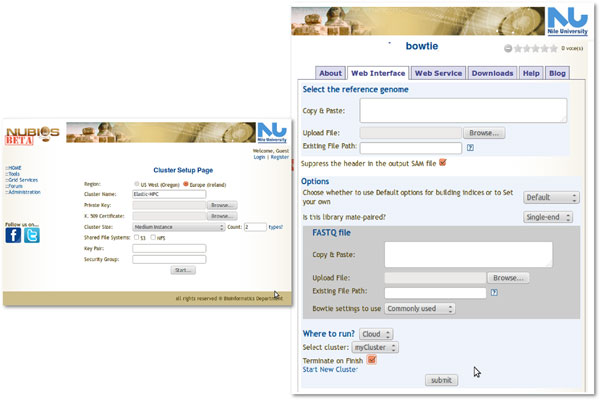
**NUBIOS server using provider use case scenario**. The NUBIOS bioinformatics server uses the provider scenario at the whole system and tool level. Left: the user set-up a cluster to instantiate the NUBIOS system on the cloud. Right: a tool page in the NUBIOS server where the user can select to run this tool on the cloud. In this case, the user has to specify an existing computer cluster in the cloud or create a new one.

Due to infrastructure limitations, the allowed job time both in BiBiServ and NUBIOS is limited to one day per user. Statistics on the server usage show that using certain tools under certain parameter settings can cause abrupt intensive load on the server side. RNAshapes [33] on BiBiServ and MegaBlast on NUBIOS are two examples of such compute intensive tools. Therefore, it would be a good solution to delegate such computations to the cloud, while running other non-intensive parts on the local infrastructure. NUBIOS and BiBiServ use the bypassed provider scenario presented in this paper, both at the system and tool level. *elasticHPC *is used for implementing both scenarios. At the former level, the user specifies an instance of the system in the cloud. This is enabled by accessing the cluster set-up page. At the latter level, the user accesses a tool page and selects that this tool is executed on the cloud. The user is also prompted to specify a cloud cluster details. Figure 7 shows screen shots from the NUBIOS web-server for both levels of use. For both scenarios, the user is then forwarded to the system or tool page in the cloud machine, where computation takes place. The *elasticHPC *image is used to create the machine instances because it includes the tool set of both NUBIOS and BiBiServ. These options clearly enable personalized services and relieve the original infrastructure from abrupt computational loads.

## Conclusions

In this paper, we introduced a set of use case scenarios to provide bioinformatics services based on cloud computing model. Our scenarios consider both the technical and financial issues. We introduced *elasticHPC *package and library to manage high performance cloud computing infrastructure with additional features to support the proposed use case scenarios. Our demos and experiments in this paper show the efficiency of cloud model in supporting computation at affordable price.

The use of *elasticHPC *is not limited to BiBiServ and NUBIOS; it can be used to support any other bioinformatics service provider like Mobyle [38] and GenePattern [39]. Currently *elasticHPC *is used within the Tavaxy workflow system http://www.tavaxy.org[40]. In this workflow system, the user can delegate the execution of some steps in the workflows to the cloud while other parts run on local infrastructure. The control and data flows are coordinated between the local infrastructure and cloud instances based on functions of *elasticHPC*.

Our current version of *elasticHPC *is limited to computer clusters. We can support other high performance computing options like GPUs, but we delayed this step because there are no bioinformatics packages so far supporting it at the production level.

Cost prediction at the tool level was suggested for BLAST [41]. This step is useful to add more abstraction to the service, in which the user can ask for faster computation time and the infrastructure is configured automatically to satisfy this requirement.

Currently *elasticHPC *is limited to AWS and to Linux environment. In future versions, we will extend it to include other providers and Windows operating systems.

*elasticHPC *is useful for educational purposes to support courses for parallel programming and advanced data processing, where students can use the package to directly build clusters in the cloud and use it to test their parallel programs and scripts. With the free start-up package of AWS, the students can use the micro instances as the types of the cluster nodes. With the job-submission interface, the students can install new tools and re-configure the machines according to the course needs.

## Availability and requirements

**Project name**: *elasticHPC*.

**Project home page**: http://www.elastichpc.org.

**Operating system(s)**: Linux.

**Programming language**: Python, C, Java script, HTML.

**Other requirements**: Compatible with the browsers FireFox, Chrome, Safari, and Opera. See the manual for more details.

**License**: Free for academics. Authorization license needed for commercial usage (Please contact the corresponding author for more details).

**Any restrictions to use by non-academics**: No restrictions.

## Competing interests

The authors declare that they have no competing interests.

## Authors' contributions

All authors contributed to theoretical developments which form the basis of the usage scenarios and *elasticHPC*. ME and MA developed *elasticHPC*. All authors wrote and approved the manuscript.
